# Preparedness of Public Hospitals for the Coronavirus (COVID-19) Pandemic in Lahore District, Pakistan

**DOI:** 10.7759/cureus.22477

**Published:** 2022-02-22

**Authors:** Afshan Shahid, Taskeen Zahra, Rabiah Mahwish, Syed M Ali Abbas Zaidi

**Affiliations:** 1 Community Medicine, Services Institute of Medical Sciences, Lahore, PAK; 2 Community Medicine, District Health Authority, Lahore, PAK

**Keywords:** novel coronavirus, resource shortage, preparedness, public hospitals, health system

## Abstract

Background

Pakistan reported more than a million cases during the coronavirus disease 2019 (COVID-19) pandemic, shuffling the already resource-constrained health system that is known for its high vulnerability and lack of adaption.

Objective

To find out the level of preparedness of public hospitals for the novel COVID-19 pandemic in Lahore district.

Methods

A descriptive cross-sectional study was conducted from April to July 2021 among all 18 public hospitals under Specialized Health Care and Medical Education (SHC&ME) in Lahore by administering World Health Organization (WHO) and SHC&ME modified and pre-tested interviewer based and observation checklist. The level of preparedness was assessed for 11 domains, and each domain was scored as a dichotomous variable (Yes and No). Hospital preparedness was labeled as ‘acceptable,’ ‘insufficient,’ and ‘unacceptable.’ Descriptive statistics were run by using SPSS version 26 (IBM Corp., Armonk, NY), and data are presented in the form of tables and bar graphs.

Results

Out of 18 hospitals, only three (17%) had an acceptable level of preparedness for COVID-19 (>70%). An unacceptable level of preparedness (<35%) was seen in one hospital (5%). Fourteen hospitals (78%) were insufficiently prepared (35-70%).

Conclusion

The study highlights the suboptimal preparedness in 83% of the public hospitals with a consistent pattern of deficiencies in surge capacity, logistics and resource management, essential services, including diagnostics, infection prevention, and control.

## Introduction

Hospitals have always been centers of health care services, and their role is highlighted during calamities. Whether pandemics are classified as disasters or not is still debatable, but it is an established fact that they lead to an increase in the spread of disease. Affecting large populations worldwide with an irregular and abrupt increase in patients’ demands that affect hospital capacities [1-2]. Just like any other disaster preparedness, hospitals need to be prepared for epidemics and pandemics. Nowadays, an overwhelming majority of the world is fighting against the pandemic caused by coronavirus disease 2019 (COVID-19), which manifested itself through illness, death, economic recession, and impeding health systems. This health crisis has shut down global economies, indicating how health care has become inseparable from economic growth and governance systems [2-3].

South Asia is among the world's poorest and most populous in Asia. This region has the lowest doctor:population ratio and very weak health systems [4], which further become vulnerable with this pandemic [5]. Regionally, Pakistan ranked third in the Global Health Security Index with a score of 35.5/100. The poor score indicates the country's lack of an emergency response and preparedness system. Globally, the country ranked 167th out of 195 countries for health indicators' performance. Like many countries, unprepared Pakistan reported 930,511 cases since the start of the pandemic [6].

Cases from the community show high transmission, indicating that treatment centers and hospitals must prepare as per the standards [7]. Hospitals work through coordinated processes that require effective management and uninterrupted supply lines [8]. The hospitals struggle to carry out necessary functional activities in the constraint capacity when the exigency of the burden health system demands it [9]. The already resource-constrained healthcare system was reconfigured to improve its ability to handle unpredictable large-scale health crises like COVID-19 while remaining sustainable.

Hospital preparation with resource management, capacity building, surveillance, and communication is the major way to prevent the further spread of the pandemic [8]. Preparedness of hospitals for COVID-19 in many South Asian countries shows limitations in backup plans, personal protective equipment (PPE), and lack of isolation centers [5]. Underestimations, insufficient preparation, and lack of communication lead to a saturation of the health system and testing strategy that is deemed an indicator of poor preparedness [10]. The lack of governmental standard operating procedures (SOPs) tends to burden the already flimsy health care system of the country [11].

Assessment of the baseline preparedness of the hospitals is essential to understand the gaps in the containment of the COVID-19 pandemic. To date, no work has been published to find the hospitals’ preparedness and implementation of these SOPs, which are deemed as key to success in the ongoing pandemic. Keeping this background knowledge, the following survey was taken up to study the preparedness of the hospitals and institutes under Specialized Healthcare and Medical Education Department (SHC&ME) Lahore and to find out their level of preparedness for the COVID-19 pandemic in Lahore district.

## Materials and methods

A descriptive cross-sectional study was carried out in Public Hospitals under the Specialized Health Care and Medical Education Department (SHC&ME) of Lahore district having a current population of 11,126,285. The total duration of this study was three months from July-September 2021. All 18 ‘specialized institutes’ (Services Hospital, Punjab Institute of Cardiology, Jinnah Hospital, Punjab Institute of Mental Health, Lahore General Hospital, Gulab Devi Hospital, Institute of Child Health, Mayo Hospital, Sheikh Zayed Hospital, Lady Aitchison Hospital, Lady Wellington Hospital, Main Munshi Teaching Hospital, Said Metha Hospital, Nawaz Sharif Hospital Yaki Gate, Kot Khawaja Saeed Hospital, Punjab Dental Hospital, Govt. Teaching Hospital Shahdara, and Sir Ganga Ram Hospital), according to the list provided by the department (SHC&ME) were included in the study. Tertiary care and specialized hospitals falling under the umbrella of SHC&ME are termed ‘Specialized Institutes’ by the Government of Punjab and are referred to as ‘Public Hospitals’ in this study. Data collection comprised a checklist from the WHO COVID preparedness manual [10] and the guidelines available at the official website of the Ministry of National Health Services and SHC&ME [12-13] that are mandatory for COVID management in hospitals.

Hospitals’ preparedness was assessed under 11 domains (referred to as D), namely, incident management system (D1), surge capacity (D2), infection prevention and control (D3), case management (D4), human resource (D5), continuity of essential health services (D6), surveillance (D7), communication (D8), point of entry (D9), laboratory services (D10), and logistics and essential support services (D11). Each domain for preparedness was scored from 0 (‘No’ according to guidelines) to 1 (‘Yes’ according to the guidelines). A total score of 224 was calculated for all domains combined (112 * 2 items).

Liaison with District Health Authority (DHA) was made. The District Health Authority is a government-based administrative body that comes under the umbrella of the Directorate General of Health Services (DGHS), and it provides support in responding to emergency health and medical issues [14]. One doctor from DHA was also included in the research team to smoothen the contact and meetings with the medical superintendents of each hospital. The hospitals’ medical superintendents were contacted and a team of two researchers visited the hospital in person to explain the purpose and objectives of the study. The hospitals’ focal persons scheduled the visits with due permission of the hospital authorities.

The data were entered, cleaned, and analyzed using Statistical Package for Social Sciences (SPSS) version 26 (IBM Corp., Armonk, NY). Descriptive statistics were analyzed in the form of frequencies and percentages for qualitative variables. The total score was aggregated and mean and standard deviations were calculated for preparedness. The data were presented in bar charts and tables. The level of preparedness was categorized as unacceptable for hospitals scoring 78 out of 224 (<35%), insufficiently prepared for hospitals scoring 35-157 out of 224 (35%-70%), and acceptable preparedness for hospitals scoring >158 (>70%) [7]. Individual preparedness categorization for each domain was carried out on the above-mentioned cut-offs.

Ethical considerations

Ethical approval was obtained from the Institutional Review Board of Services Institute of Medical Sciences (SIMS), Lahore (Ref No: IRB/877/SIMS).

## Results

A total of 18 public hospitals under the SHC&ME within Lahore were assessed for their preparedness of COVID-19. See Table 1.

**Table 1:** Performance of public sector hospitals for each assessed domain in Lahore

Hospital	Domain (D) & Score											Total Score (224)
	D1 (10)	D2 (16)	D3 (54)	D4 (20)	D5 (26)	D6 (8)	D7 (25)	D8 (13)	D9 (13)	D10 (25)	D11 (14)	
Hospital 1	5	5	25	13	15	5	12	3	3	11	8	105
Hospital 2	6	10	25	8	15	5	10	8	8	13	10	118
Hospital 3	9	15	30	16	18	6	24	8	8	22	10	166
Hospital 4	5	00	22	1	10	3	9	6	6	9	8	79
Hospital 5	8	13	44	16	18	5	14	6	6	22	10	162
Hospital 6	9	3	40	14	14	4	18	10	10	13	10	145
Hospital 7	9	8	39	15	14	4	21	8	8	9	8	143
Hospital 8	9	15	46	20	22	6	22	10	10	22	11	193
Hospital 9	9	14	21	17	20	6	20	8	8	15	11	149
Hospital10	6	9	38	11	18	5	19	8	8	15	8	145
Hospital 11	6	9	28	15	18	5	13	9	9	13	8	133
Hospital 12	5	9	20	10	15	4	16	7	7	14	6	113
Hospital 13	5	9	21	15	14	4	24	9	9	8	6	124
Hospital 14	6	9	36	17	14	4	24	8	8	9	6	144
Hospital 15	5	9	37	13	14	4	22	9	9	16	6	144
Hospital 16	5	0	16	2	14	4	9	6	6	2	6	70
Hospital 17	5	3	9	9	14	4	12	6	6	15	6	89
Hospital 18	6	11	5	7	15	5	16	8	8	14	8	103

The numbers allocated to each domain were allocated according to the requirements that were essential from the SHC&ME department to manage COVID-19 in each facility. The performance of these hospitals in these 11 domains was as follows:

Incident management system (D1)

This domain was a fountainhead of all the assessments carried out for preparedness for the COVID-19 pandemic and included disaster planning of hospitals, presence of a rapid response team, designating a COVID-19 response focal person or committee, and their liaison with the district health authority. Thirteen (72%) hospitals had an insufficient level of preparedness in this domain and only five (28%) hospitals were ‘acceptably’ prepared. Although all hospitals had a management plan, a focal person, and an isolation area designated for the suspected or diseased cases of COVID-19, more than one quarter (39%) were reluctant to proceed. The mean score for 18 hospitals in D1 was 6.5.

Surge capacity (D2)

All hospitals were assessed based on the availability of mechanical support (ventilators) and intensive care units and the increased capacity of hospital beds for indoor patients. Only four out of 18 (22%) had ‘acceptable’ preparation, the remaining 14 out of 18 (78%) of the hospitals had ‘insufficient’ preparation. The poor preparation in this category depicts a catastrophe during the increase in COVID-19 cases.

Infection prevention and control (IPC) (D3)

Indoor and outdoor units, intensive care units, and emergency departments were visited for D3. Observations were made and questions were asked from the focal person regarding IPC. Except for four (22%), all hospitals lacked a special filter clinic or triage area for respiratory symptoms. No separate COVID-19 patient transit was observed in hospitals except one; however, separate wards were designated in all except three (17%), which were not entertaining any COVID-19 patients at all.

Five out of 18 (28%) had an acceptable level of preparation. Three (17%) fell into the ‘unacceptable’ preparedness level while 10 out of 18 (55%) were ‘insufficiently’ prepared. The designation of special donning and doffing areas was seen in all hospitals except three; however, efforts were made in all facilities towards IPC. All the hospitals stressed the availability of personal protective equipment (PPE) (mask, gown, gloves, and eye shields). Uncertainty over the continuous availability of PPEs was observed, as they were either donated or self-purchased. The mean hospitals score for this domain was 28.

Case management (D4)

Five out of 18 (28%) had an ‘acceptable’ level of preparedness. Two out of 18 (11%) were ‘unacceptably’ prepared while 11 out of 18 (67%) hospitals were insufficiently prepared. Health care workers in most of the hospitals were aware of the standard guidelines for COVID-19 management despite poor logistics and poor incident management systems in 28% of hospitals. The mean score for 18 hospitals was 12.

Human resource (D5)

For this domain, the majority of the hospitals 17 (94%) were sensitive to their staff strength and training for the COVID-19 pandemic. The facility of contact tracing of infected health care providers was also available in these 18 hospitals. One hospital was referring such health care providers and had no plans to enhance its staff strength and training. Lack of recognition and special incentives were still points of concern among the workers. The mean score for 18 hospitals was 15.6.

Continuity of essential health services (D6)

The ability of a hospital to prioritize and maintain the services that should be provided, along with the required resources, was studied in this domain. Only three (17%) hospitals were prepared; the remaining 15 (83%) were insufficiently prepared. Three (17%) referred their COVID-19 cases to other hospitals and provided uninterrupted routine services. Although the remaining hospitals had devised their priority plans and their identification and requirement of resources for the provision of essential health services, this was still under process. The mean score for 18 hospitals was 15.6.

Surveillance (D7)

In this domain, 10 out of 18 (55%) hospitals scored from 35%-70%. None of the hospitals was in the ‘unacceptable’ level of preparedness; eight out of 18 (45%) had an ‘acceptable’ level of preparedness. Hospitals were regularly collecting data of COVID-19 patients and sharing this data with the district health authority and COVID-19 dashboard. The other components of this domain such as training regarding surveillance and response for staff members and nominating an epidemiologist were lagging. The mean score for this domain was 16.9.

Communication (D8)

Risk communication infographics were displayed in all hospitals. Further, there was an availability of a help desk for patients and their attendants. No individual or official collaboration with social and print media was noted in any of the hospitals. The mean score for 18 hospitals was 7.6.

Point of entry (D9)

All hospitals denied entry to people without wearing a mask but fever scanning via an infrared thermometer was observed only in 12 (67%) hospitals. The mean score for 18 hospitals was 7.6.

Laboratory services (D10)

The biosafety level of the laboratory, the nature of the sample collected for COVID-19-suspected cases, sample handling and transportation, provision of a viral medium, and a cold box and its disinfection were the key components of this domain. Only three out of 18 (17%) hospitals had Biosafety Level 3 labs, the rest of the hospitals were sending their samples to government-nominated institutes. However, a proper chain of sample collection, transportation, and disinfection was observed. Thirteen hospitals (72%) had an ‘insufficient’ level of preparedness and two (11%) had ‘unacceptable.’ The mean score for 18 hospitals was 13.4.

Logistics and essential support services (D11)

This domain included the continuation of the regular hospital services besides dealing with COVID-19 patients. The mean score for 18 hospitals was 8.1. It was noted that 12 out of 18 hospitals (66.6%) had a score of less than the mean, indicating their inability to cope in the estimation of additional supplies. Only 34.4% of hospitals had the capability to anticipate the impact of the infection and prepare accordingly. The hospitals stressed the need for a continuous supply of logistics by the government, as they were working in an uncertain environment, and this again raises the concern of resource limitation and over-burdening of the hospitals that are performing well.

Provision of a hand-washing facility indoors was noticed in 15 (83%) hospitals and the practice of regular disinfection was observed in the outdoors of 16 (89%) hospitals. Six (33%) hospitals did not adopt any method to maintain social distance in the outpatient department, which was found to be over-crowded and had long queues at the receipt counters.

Among 18 hospitals, only three (17%) scored an acceptable level of preparedness for COVID-19 (>70%), scores ranging from 193 to 162 out of 224; one (5%) hospital had an ‘unacceptable’ score at the 70/224 level (<35%). An insufficient level of preparedness was observed in 14 out of 18 hospitals (78%), who scored below 35%-70% with a score ranging from 79-148.

## Discussion

The current pandemic has taken the world by storm and requires meticulous preparedness in health care settings. A well-prepared public hospital can reduce the demand for specialized healthcare and plays an important role in minimizing the spread of the pandemic [15-16].

The COVID-19 pandemic unearthed inequities and challenges that require scaling up strategic preparedness for future pandemics [6]. It was found that only three (17%) reached an acceptable level of preparedness. The performance of one hospital (6%) was unacceptable while the remaining 14 (77.7%) hospitals had insufficient preparation for the COVID-19 outbreak in different domains out of the total 11 domains assessed. Tiruneh and his colleague showed that 37.5% of hospitals in North-West Ethiopia had an ‘unacceptable’ level of preparedness, 12.5% had an acceptable level, and the remaining 50% of hospitals had insufficient levels of preparedness [7]. The public hospitals' preparedness score was 129 ± 32, which is better than the mean score of health care facilities of Ethiopia where the mean score was 70.3 ± 21.6 [17]. In the same study, it was concluded that 73.9% of the health centers still had insufficiencies and fell in the “work to do” category; conversely, the score of no health facility fell below this [17]. Zia and his colleagues predicted poor performance of the local health system during the crisis, pertaining to sub-standard execution of the coordination, operational and procedural aspects [18]. It is worth noting that Pakistan’s strategic preparedness and response to COVID-19 falls in Level 2 (<40%), which again points to deficiencies in strategic preparedness [19]. It is a dilemma that the health care delivery system is mostly ‘disease treatment ‘oriented and little emphasis is placed on planning and preparedness [20].

The COVID-19 pandemic is a complex, unprecedented public health crisis requiring efficient coordination of various activities. In this aspect, the incidence management system (IMS, also referred to as the incidence command system or ICS [21]) applies the concept of a unified approach. Sixty-one point two percent (61.2%) of the hospitals scored above 50% in the IMS domain. It is worth noting that none of the hospitals had a score below 50%, indicating the realization of the importance of unison perspective, which can assist hospitals in better management during the surge. The results were slightly better than Tiruneh et al. who showed that 50% of public hospitals in North Ethiopia scored 50% in this domain with a mean score of 6.7 [7].

Resource management (including human resource, logistics, and essential support services), coordination, communication, and information management guided by standardization are important pillars in crisis preparedness. Public hospitals were handling human resources quite well but logistics and support services were beyond their grip. This clearly shows that this pandemic needs a multidisciplinary approach where the government needs to ensure a continuous supply chain. A tight relationship exists between case management, continuity of health services, surveillance, communication, logistics and essential support services, laboratory services, and the institutional structure and duration of the operation. McMahon also argued that a lack of logistics affects the provision of services in the management of the COVID-19 pandemic and is bad news for low-income countries [22].

Besides the novelty and adequacy of a hospital, human resource plays a crucial role. Restructuring of the health care worker force (HCWF) was observed. COVID-19 case management training programs were conducted in all hospitals. It was noted that no measures were taken by any hospital for the mindfulness and wellness of HCWF. A lack of recognition of services by the government and public was a point of concern for HCWs in all hospitals. Zahid et al. found that 97.5% of the HCWs exhibited high burnout in aspects of depersonalization during their COVID-19 duties, with 83.3% of them worried about the non-availability of PPE. The HCWs were exhausted emotionally and were experiencing depersonalization [23]. Gupta and Federman argued that re-organizing the duty hours along with happy endeavors, teamwork, enhancement of existing resilience, and appreciation can play a critical role in their mental health [24].

Sustaining the health services and tackling the surge during the COVID 19 pandemic is a point of concern for all nations let alone low and middle-income countries. During the COVID-19 pandemic, the number of patients that required ventilator support outnumbered the available intensive care unit (ICU) beds [25-26]. Less than a quarter (22%) of the surveyed hospitals had an ‘acceptable’ level of preparedness; Barasa in Kenya also found similar results [27]. No published data for South Asia was discovered in this regard.

Madiha and her coworker in a cross-sectional survey of intensive care units across the country showed 0.7 beds per 100,000 population with a 1:1 ventilator to bed ratio in only 52% of hospitals and the availability of senior clinicians in only 12.1% of the hospitals [28]. The hospital and ICU bed surge capacity varied substantially across the country, predicting the burdening of functioning units. The lack of preparedness of hospitals in the domains of surge capacity and logistics and the inability to continue essential services foresees the exhaustion of human and material resources in 17% of the hospitals that were well prepared.

Due to high transmission, WHO stressed good prevention and control of the disease. This containment depends immensely on the principles of rapid identification, prevention, and control, followed by patient isolation, laboratory diagnosis, and contact tracing [29]. Screening and triage, application of standard precautions for all patients, and the implementation of administrative, environmental, and engineering measures are components of the five-pronged strategy devised by WHO for IPC. Only 28% of the hospitals had acceptable levels of preparedness for IPC and case management. The results were slightly better than other low-income countries where 12.5% and 21.9% of hospitals had sufficient IPC, including PPE [22]. It can be due to the fact that the HCWF did not rely solely on the government for it and worked in its full capacity to ensure IPC, including at the point of entry. Poor IPC and poor supply of PPEs was the point of concern for 83.3% of frontline workers in Karachi, causing them emotional stress and anxiety [23].

Five (28%) hospitals were referring COVID-19 suspects or cases to other facilities despite having trained staff. This clearly shows that these hospitals can share the burden of other institutes by managing their logistics, engineering measures, and laboratory services and can otherwise choke the public hospitals that are working at their full capacity. Zia-ul-Haq also confirmed unacceptable levels of preparedness of public hospitals in disaster-prone provinces of the country in 2019, in the management of situations involving mass fatalities, emergency telecommunications, and capacity for case management [18].

Surveillance and rapid identification of cases are highlighted features in prevention and control, which help in the identification of trends, course of action, and target interventions [29]. No hospital had an ‘unacceptable’ level of preparedness; however, more than half had an ‘insufficient’ score. This was due to the mandatory activity of data sharing with the government and its agencies. However, hospitals still lacked epidemiologists, contact tracing efforts, and surveillance training programs. The hospitals that outperformed others were covering these aspects. Ayele et al. showed better results where 78.3% of hospitals had a focal person and emergency committees but Tiruneh et al,'s results showed that only one hospital out of eight had an ‘acceptable’ level of preparedness in D7 [7,17]. However, hospitals still performed better keeping in mind that Pakistan ranked 98th among 195 in GHS for the detection category [5].

Limitations

The study was not without limitations. The inability to include primary and secondary healthcare facilities and private hospitals due to resource constraints leaves unaddressed ground realities that can haze the actual picture. Taking into account the geographical catchment of the hospitals and correlating the hospital preparedness with performance during the peaks of the wave were beyond the objectives of this study. The information provided by the focal person and HCWF at the spot was taken as authentic and was not cross-checked.

In spite of the limitations, the study is one of a kind and accounts for public hospital preparedness in a densely populated metropolitan city of Pakistan. It reciprocates the governments' and HCWs' efforts at all levels and identifies the gaps for improvement and future research.

## Conclusions

The current global economic and health situation is the point of concern for low and middle-income nations with fragile health systems and low-quality health care services. Pandemic preparedness requires the management of all resources, including humans, equipment, materials, and information. Resources are always a constraint in public hospitals, and the situation becomes more critical in cases of such catastrophes. Hospital preparedness is an essential component of an emergency plan that can counter the impact of large-scale epidemics. Therefore, evaluating organizational readiness is an essential step in this planning process, especially in pandemics like COVID-19, which has fluctuating hospital demands due to multiple disease waves.

This study assessed the hospital preparedness of public sector hospitals against the COVID-19 pandemic using the checklist prepared from national guidelines. These components are concerned with the patient surge, infection monitoring and control, human resource, case management, communication, supply chain management, laboratory services, surveillance, and essential services. Under these domains, a total score was calculated for each hospital's weight of these subcomponents; a preparedness index for each hospital was obtained. The index is specifically computed individually for each hospital in each domain to compare the overall preparedness among different hospitals. This helps decision-makers in specialized health care in observing the readiness level of the hospital from 11 different viewpoints. The study highlighted the suboptimal preparedness in 83% of public hospitals with a consistent pattern of deficiencies in surge capacity, logistics and resource management, essential services, including diagnostics, and infection prevention and control, which require a special focus of the providers to cater for the resurge of the pandemic in future.
